# Anti-addiction drug ibogaine inhibits voltage-gated ionic currents: A study to assess the drug's cardiac ion channel profile^☆^

**DOI:** 10.1016/j.taap.2013.05.012

**Published:** 2013-12-01

**Authors:** Xaver Koenig, Michael Kovar, Lena Rubi, Agnes K. Mike, Peter Lukacs, Vaibhavkumar S. Gawali, Hannes Todt, Karlheinz Hilber, Walter Sandtner

**Affiliations:** a Center for Physiology and Pharmacology, Department of Neurophysiology and -pharmacology, Medical University of Vienna, 1090 Vienna, Austria; b Center for Physiology and Pharmacology, Institute of Pharmacology, Medical University of Vienna, 1090 Vienna, Austria

**Keywords:** hERG, human Ether-à-go-go-Related Gene, 18-MC, 18-Methoxycoronaridine, AP, action potential, Anti-addiction drug, hERG potassium channels, Ibogaine, 18-Methoxycoronaridine, QT interval prolongation, Voltage-gated ion channels

## Abstract

The plant alkaloid ibogaine has promising anti-addictive properties. Albeit not licenced as a therapeutic drug, and despite hints that ibogaine may perturb the heart rhythm, this alkaloid is used to treat drug addicts. We have recently reported that ibogaine inhibits human ERG (hERG) potassium channels at concentrations similar to the drugs affinity for several of its known brain targets. Thereby the drug may disturb the heart's electrophysiology.

Here, to assess the drug's cardiac ion channel profile in more detail, we studied the effects of ibogaine and its congener 18-Methoxycoronaridine (18-MC) on various cardiac voltage-gated ion channels. We confirmed that heterologously expressed hERG currents are reduced by ibogaine in low micromolar concentrations. Moreover, at higher concentrations, the drug also reduced human Na_v_1.5 sodium and Ca_v_1.2 calcium currents. Ion currents were as well reduced by 18-MC, yet with diminished potency. Unexpectedly, although blocking hERG channels, ibogaine did not prolong the action potential (AP) in guinea pig cardiomyocytes at low micromolar concentrations. Higher concentrations (≥ 10 μM) even shortened the AP. These findings can be explained by the drug's calcium channel inhibition, which counteracts the AP-prolonging effect generated by hERG blockade. Implementation of ibogaine's inhibitory effects on human ion channels in a computer model of a ventricular cardiomyocyte, on the other hand, suggested that ibogaine does prolong the AP in the human heart. We conclude that therapeutic concentrations of ibogaine have the propensity to prolong the QT interval of the electrocardiogram in humans. In some cases this may lead to cardiac arrhythmias.

## Highlights

• We study effects of anti-addiction drug ibogaine on ionic currents in cardiomyocytes.
• We assess the cardiac ion channel profile of ibogaine.
• Ibogaine inhibits hERG potassium, sodium and calcium channels.
• Ibogaine’s effects on ion channels are a potential source of cardiac arrhythmias.
• 18-Methoxycoronaridine has a lower affinity for cardiac ion channels than ibogaine.

## Graphical abstract

**Figure:**
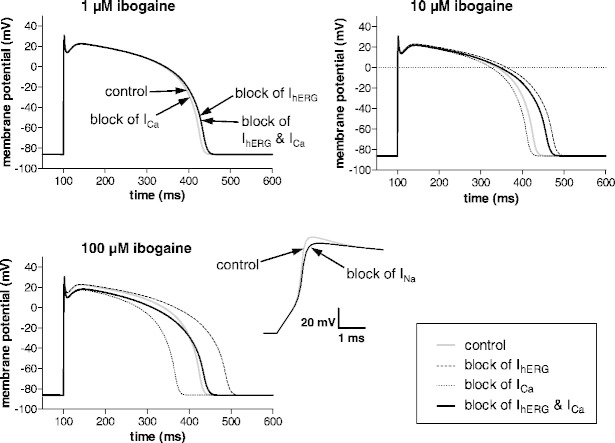

## Introduction

Ibogaine, an indole alkaloid derived from the root bark of the African shrub *Tabernanthe iboga*, has received much attention because of its promising “anti-addictive actions” (for reviews see Alper, 2001; Maciulaitis et al., 2008). Thus, in animals, ibogaine attenuates opioid withdrawal signs and reduces the self-administration of a variety of drugs including opioids, cocaine, nicotine, and alcohol (Alper, 2001; Glick and Maisonneuve, 1998). In addition, ibogaine hampers responses that are associated with addiction, such as dopamine release in the *nucleus accumbens* of the brain (Benwell et al., 1996; Maisonneuve et al., 1991). Ibogaine's precise mechanisms of action remain unclear, but its effects may emerge from complex interactions with multiple neurotransmitter systems. Accordingly, ibogaine interacts with numerous different cellular and molecular targets, e.g. neurotransmitter transporters, opioid receptors, sigma receptors, glutamate receptors, and nicotinic receptors (Alper, 2001; Glick and Maisonneuve, 1998; Glick et al., 2000; Maciulaitis et al., 2008).

Ibogaine has a long history of use as a medicinal and ceremonial agent in West Central Africa. Besides its own psychoactive properties, anecdotal evidence suggests that ibogaine also acts as an anti-addictive in humans. Thus, intake of this alkaloid alleviates drug craving and impedes relapse of drug use (Alper, 2001; Maciulaitis et al., 2008; Mash et al., 1998). In spite of its status as a banned substance in the U.S. and some European countries, ibogaine is legal in most of the world, and, although not licenced as a therapeutic drug, is currently used as an anti-addiction drug in alternative medicine (Alper et al., 2008; Vastag, 2005).

Because ibogaine has a complex pharmacology and is known to interact with numerous different targets (see above), its potential to generate adverse effects is significant. Besides the expected neurotoxic actions (e.g. (Alper, 2001; Maciulaitis et al., 2008; Molinari et al., 1996; O'Hearn and Molliver, 1993; Xu et al., 2000)), ibogaine also affects the cardiovascular system. In rats, high doses of ibogaine decreased the heart rate without altering blood pressure (Binienda et al., 1998; Glick et al., 2000). This finding is consistent with anecdotal reports in humans that ibogaine slows the heart rate (Alper, 2001; Glick et al., 2000; Maciulaitis et al., 2008). Alarming are several cases of sudden deaths after ibogaine use with unclear cause (Alper et al., 2012; Donnelly, 2011), which have been hypothesised to be related to cardiac arrhythmias (Alper et al., 2012; Hoelen et al., 2009; Maas and Strubelt, 2006). Due to concomitant medications used and comorbidities present in the patients described in these cases, however, it is unclear whether ibogaine alone or in combination with other factors may contribute to the clinical adverse findings. Recently several cases of ibogaine-associated QT interval prolongation and arrhythmias were reported (Hoelen et al., 2009; Paling et al., 2012; Pleskovic et al., 2012). In a first attempt to elucidate the mechanism(s) by which ibogaine may account for the described clinical observations, we recently (Koenig et al., 2012) tested the drug's propensity to inhibit human ERG (hERG, IK_r_) potassium currents. hERG channels are crucial for the repolarisation phase of the cardiac action potential (AP), and hERG channel blockade by drugs is considered the most common reason for drug-induced QT interval prolongation, which can be associated with an increased cardiac arrhythmia risk (Redfern et al., 2003; Sanguinetti and Tristani-Firouzi, 2006). Indeed, we found that ibogaine reduces hERG currents (IC_50_, 4 μM) at concentrations similar to the drug's affinities for several of its known targets in the brain (Koenig et al., 2012). Thus our finding matches with the described reports of QT interval prolongation after ibogaine intake (Hoelen et al., 2009; Paling et al., 2012; Pleskovic et al., 2012).

Here, to study in more detail the possibly harmful impacts of ibogaine on the heart's electrophysiology, we explored the drug's effects on the function of various cardiac voltage-gated ion channels. Therefore, human ion channels that contribute significantly to the action potential (hERG potassium channels, hNa_v_1.5 sodium channels, and hCa_v_1.2 calcium channels) were heterologously expressed in TSA-201 cells. In addition, ibogaine's effects on the cardiac AP were assessed in experiments on ventricular cardiomyocytes derived from adult guinea pig hearts, and in simulations using a computer model of a human ventricular cardiomyocyte. In an effort to estimate margins of safety for this drug between the ion channel potencies in vitro and human clinical exposure, we determined human plasma protein binding of the drug. Moreover, we also tested its synthetic congener 18-Methoxycoronaridine (18-MC), which exhibits anti-addictive effects but is believed to be less toxic (Glick et al., 1996, 2000, 2001, 2011).

## Materials and methods

The study complies with the rules of the University Animal Welfare Committee. All of the procedures were conducted in accordance with the European Community Council Directive for Care and Use of Laboratory Animals.

### Culture and transfection of TSA-201 cells

TSA-201 cells (American Type Culture Collection (ATCC, Manassas, VA, USA)) were propagated in a Dulbecco's modified Eagle's medium (DMEM, Invitrogen, Vienna, Austria) containing 10% foetal bovine serum (FBS), 50 U/ml penicillin, and 50 U/ml streptomycin, and were incubated at 37 °C in a humidified incubator with 5% CO_2_. Cells were transfected with pCEP4-plasmid (0.7 μg per 3.5 cm dish) containing the coding sequence for the human cardiac hERG potassium channel, or pGEM3-plasmid (1–2 μg per 3.5 cm dish) containing the coding sequence for the human cardiac hNa_v_1.5 sodium channel. For hCa_v_1.2 calcium current expression, an equimolar ratio of cDNA (1 μg per 3.5 cm dish) encoding for hCa_v_1.2 α_1_ subunit (77-pcDNA3) together with auxiliary rCa_v_β_3_ and rCa_v_α_2_δ_1_ subunits was used. 77-pcDNA3 (Soldatov et al., 2000) was used in this work with the permission of Dr. Nikolai M. Soldatov (Humgenex, Inc., Maryland, USA). Cotransfection with pEGFP-C1-plasmid (0.02 μg) encoding green fluorescent protein (GFP) allowed the identification of successfully transfected cells. For transfection of hERG, hNa_v_1.5 and hCa_v_1.2, ExGen 500 (Fermentas, St. Leon-Rot, Germany) and FuGENE (Promega, Mannheim, Germany) were used according to the manufacturer's protocol.

### Isolation of adult guinea pig ventricular cardiomyocytes

Ventricular cardiomyocytes from adult female Dunkin-Hartley guinea pigs (200–400 g) were isolated using a Langendorff setup following procedures previously described (Koenig et al., 2011).

### Drug sources and preparation of stock solutions

Ibogaine hydrochloride originated from Sacrament of Transition (Maribor, Slovenia). 18-Methoxycoronaridine (18-MC) racemate was purchased from Obiter Research (Champaign, IL, USA). Both drugs were dissolved in 0.1% HCl, and stock solutions with various concentrations in the range of 1 μM to 30 mM (upper solubility limit) were prepared. These stocks were stored at − 20 °C. On the day of the experiment, stock aliquots were freshly diluted 1:100 with the respective bath solutions to obtain final drug concentrations (range between 0.01 and 300 μM). The drug-free control bath solutions contained the same amount of HCl than the experimental solutions. Because of limitations in drug solubility in our bath solutions, we refrained from using higher drug end concentrations than 300 μM. E-4031 and isradipine were purchased from Sigma-Aldrich (Vienna, Austria).

### Electrophysiological studies using the whole cell patch clamp technique

A detailed description of the electrophysiological recordings is given in our earlier work (Mille et al., 2009; Zebedin et al., 2008). Ionic currents were recorded from TSA-201 cells 24–48 h after transfection, and from adult cardiomyocytes up to 12 h after preparation at room temperature (22 ± 1.5 °C), using an Axoclamp 200B patch clamp amplifier (Axon Instruments, Union City, CA). Pipettes were formed from aluminosilicate glass (A120-77-10; Science Products, Hofheim, Germany) with a P-97 horizontal puller (Sutter Instruments, Novato, CA), and had resistances between 0.8 and 2 MΩ when filled with the respective pipette solutions (see below). Data acquisition was performed with pClamp 6.0 software (Axon Instruments) through a 12-bit A-D/D-A interface (Digidata 1200; Axon Instruments). Data were low-pass filtered with 1–10 kHz (− 3 dB) and digitised at 10–100 kHz. Data analysis was performed using Clampfit 10.2 (Axon Instruments) and GraphPad Prism 5.01 (San Diego, USA) software. Rapid solution changes were performed by a DAD-8-VC superfusion system (ALA Scientific Instruments, Westbury, NY, USA).

#### hERG potassium currents

The pipette solution contained 130 mM KCl, 5 mM MgCl_2_, 5 mM K_2_-ATP, 5 mM EGTA, 10 mM HEPES (pH = 7.2, adjusted with KOH). Recordings were made in a bath solution that consisted of 137 mM NaCl, 4 mM KCl, 1.8 mM CaCl_2_, 1 mM MgCl_2_, 10 mM glucose, 10 mM HEPES, pH = 7.4 adjusted with NaOH.

#### Sodium currents

The pipette solution contained 105 mM CsF, 10 mM NaCl, 10 mM EGTA, 10 mM HEPES, pH = 7.3 adjusted with CsOH. Recordings of hNa_v_1.5 sodium channels expressed in TSA-201 cells were made in a bath solution that consisted of 140 mM NaCl, 2.5 mM KCl, 1 mM CaCl_2_, 1 mM MgCl_2_, 10 mM HEPES, pH = 7.4 adjusted with NaOH.

#### Barium currents

The pipette solution contained 145 mM Cs-aspartate, 2 mM MgCl_2_, 10 mM HEPES, 0.1 mM Cs-EGTA, 2 mM Mg-ATP, pH = 7.4 adjusted with CsOH. The bath solution contained 10 mM BaCl_2_, 145 mM TEA-Cl, 10 mM HEPES, pH = 7.4 adjusted with TEA-OH.

#### Action potential recordings

APs were recorded from guinea pig ventricular cardiomyocytes in the current-clamp mode of the whole cell patch clamp technique. APs were elicited at 1 Hz by rectangular current pulses of 4 ms duration at 125% threshold level. The pipette solution contained 10 mM NaCl, 140 mM KCl, 2 mM EGTA, 1 mM MgCl_2_, 0.1 mM Na-GTP, 5 mM Mg-ATP, 10 mM Hepes, pH = 7.2 adjusted with KOH. The cells were bathed in 140 mM NaCl, 4 mM KCl, 2 mM CaCl_2_, 2 mM MgCl_2_, 5 mM HEPES, 5 mM glucose, pH = 7.4 adjusted with NaOH.

### Data analysis and curve fitting

Curve fitting was performed with nonlinear least square regression in Prism 5.01 (GraphPad Software). Normalised hERG activation data of individual experiments were fit to a Boltzmann function: I_norm_ = 1 / (1 + exp((V_0.5_ − V) / K)) where I_norm_ is the normalised hERG tail current, V is the membrane potential, V_0.5_ is the voltage at which half maximum activation occurred, and K is the slope factor. To assess hERG channel deactivation, tail currents were fit with a double exponential function: I_tail_ = A_fast_ ∗ exp(− t / τ_fast_) + A_slow_ ∗ exp(− t / τ_slow_) + C (Clampfit 10.2), where τ_fast_ and τ_slow_ are the fast and slow time constants, A_fast_ and A_slow_ are the respective amplitudes, and C is a constant. Relative amplitudes were determined by dividing by the total amplitude (e.g. A_fast_ / (A_fast_ + A_slow_)). Current–voltage relationships for hNa_v_1.5 were fit with the function: I = G_max_ ∗ (V − V_rev_) / (1 + exp((V_0.5_ − V)/K)) where I is the current, G_max_ is the maximum conductance (for the remaining parameters see above). Concentration-response data were fit with a Hill equation: I_norm_ = 100 / (1 + (IC_50_/C)^h) where the minimal and maximal values were constrained to 0% and 100%, respectively. I_norm_ is the current (%) during drug exposure in relation to the current during drug-free conditions, IC_50_ is the concentration at 50% current inhibition, C is the drug concentration, and h is the Hill slope. For all concentration–response curves the corresponding Hill slopes were between 0.8 and 1.3, thus implicating a single drug binding site.

Statistical comparisons of data before and after drug application were performed by paired two-tailed Student's t-tests. One-way analysis of variance (ANOVA) followed by Tukey's post hoc test was used when more than two groups had to be compared. A p-value of < 0.05 was considered significant. For experiments to assess the concentration-dependence of channel inhibitions by drugs the group sizes (= number of experiments (n) performed at different drug concentrations) were expressed as ranges.

### Computer simulation of the human cardiac action potential

A detailed model of a human ventricular cardiomyocyte ((ten Tusscher et al., 2004), mid-myocardial version) was downloaded from http://www.cellml.org/models. All simulations were performed within the CellML environment (Lloyd et al., 2008) with the default parameters of the model. Action potentials were simulated with a step size of 0.1 ms and at a rate of 1 Hz. The inhibitory effects of ibogaine were implemented by adapting the ionic conductances of the model, gNa for the voltage-gated sodium current, gCaL for the voltage-gated l-type calcium current, and gKr for hERG potassium current, according to the observed experimental inhibitions by the different drug concentrations.

Throughout the manuscript data represent means ± standard deviation.

## Results

### Drug effects on human ERG potassium channels heterologously expressed in TSA-201 cells

**Fig. 1:**
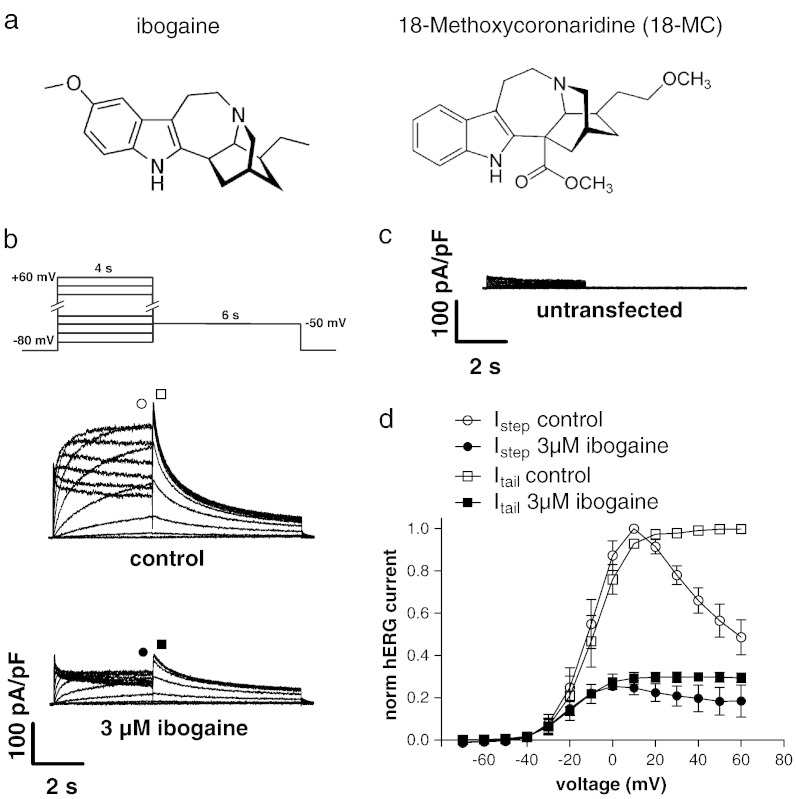
hERG potassium channel blockade by ibogaine. (a) Chemical structure comparison between ibogaine and 18-MC. (b) Typical whole cell currents through hERG potassium channels heterologously expressed in a TSA-201 cell. Currents elicited by the pulse protocol on top under control conditions, and after steady-state level was reached during superfusion with bath solution containing 3 μM ibogaine are displayed. In addition, typical endogenous currents of an untransfected TSA cell, elicited by the same pulse protocol, are shown (c). (d) Summary of the current–voltage relationships under control conditions (empty symbols) and under steady-state block with 3 μM ibogaine (filled symbols). The current amplitudes were analysed at the end of the initial 4 s test pulses (I_step_, circles), and at the peak of the tail currents (I_tail_, squares) elicited by the subsequent 6 s pulse to − 50 mV, and plotted against the applied voltage. The solid lines connect the single data points. Values represent means ± standard deviation.

We have recently reported (Koenig et al., 2012) that ibogaine concentrations in the low micromolar range inhibit hERG channels expressed in TSA-201 cells. Here, we characterised ibogaine's effects on hERG channels in more detail. In addition, we also tested the effects of its congener 18-Methoxycoronaridine (18-MC). The structures of the two compounds are compared in Fig. 1a. Fig. 1b shows typical whole cell currents through hERG channels heterologously expressed in a TSA-201 cell under drug-free control conditions, and after steady-state block with 3 μM ibogaine applied from the external side. These currents were elicited by the pulse protocol depicted on top from a holding potential of − 80 mV. It can be observed that ibogaine decreased the hERG current amplitude. Endogenous currents in untransfected TSA-201 cells were small (19 ± 9 pA/pF, Fig. 1c) and did not contribute to the overall HERG current. To further confirm the presence of functional hERG channels in our heterologous expression system, at the end of several experiments, the hERG channel blocker E-4031 was applied at a concentration of 1 μM. As expected, this drug completely inhibited the hERG tail currents in TSA-201 cells (data not shown). Fig. 1d shows a summary of the current–voltage relationships from seven experiments as shown in b. The current amplitudes were evaluated at the end of the initial depolarising voltage steps (circles) and at the peaks of the subsequent tail current (squares), under control conditions (empty symbols) and under 3 μM ibogaine (filled symbols). In the presence of ibogaine, the voltage at which half maximum activation was reached (V_0.5_) was shifted to more hyperpolarised potentials from − 9.4 ± 3.8 mV in the absence to − 18.6 ± 4.5 mV in the presence of the drug (n = 7; analysis of tail current peaks; p < 0.0001). The corresponding slope factors (K values) were not significantly different and yielded 7.5 ± 0.5 and 7.8 ± 0.7 mV, respectively (p = 0.6). The observed negative shift in V_0.5_ under ibogaine could be reversed by wash out of the drug. V_0.5_ and K two minutes after beginning of wash out amounted to − 10.1 ± 5.3 and 7.4 ± 0.5 mV, respectively (n = 4).

Finally, the tail currents elicited by the 6 s voltage step to − 50 mV (see Fig. 1b) were further analysed (seven experiments). The decay of these currents represents the kinetics of hERG channel deactivation (Chen et al., 1999; Perry et al., 2010). Double exponential fits to the tail currents revealed the following decay time constants (τ-values): τ_fast_ = 330 ± 70 ms (relative amplitude, 0.46) and τ_slow_ = 2050 ± 380 ms (0.54) in the absence, and τ_fast_ = 550 ± 250 ms (0.44) and τ_slow_ = 2990 ± 1320 ms (0.56) in the presence of the drug. A paired Student's *t*-test between control and 3 μM ibogaine for τ_fast_ and τ_slow_ yielded p < 0.05 and p = 0.05, respectively. The relative amplitudes given in brackets were unchanged. Thus, under 3 μM ibogaine, hERG channel deactivation was considerably slowed. This effect was fully reversible after washout.

**Fig. 2:**
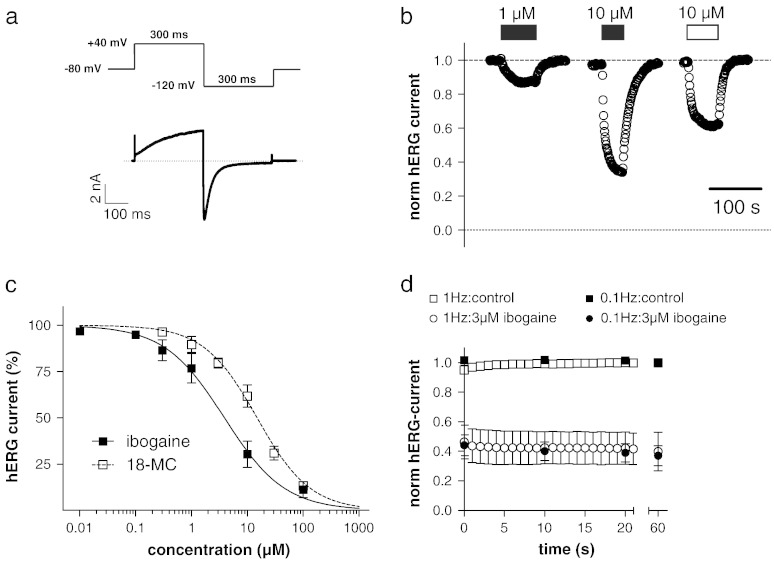
Concentration-dependence of hERG channel inhibition by ibogaine and 18-MC. (a) The pulse protocol to elicit hERG currents during a drug application experiment was applied at a frequency of 1 Hz. (b) Ibogaine (filled bars) and 18-MC (empty bar) at the indicated concentrations were applied and washed out, and the tail current peaks were plotted against time. A drug application period of 2 min was sufficient to reach steady-state block. (c) Concentration–response curves for the reduction of hERG tail currents by ibogaine (Koenig et al., 2012) and 18-MC. The pulse protocol used is displayed in Fig. 2a. The lines represent sigmoidal fits to the data points with a Hill equation. (d) hERG current reduction during 60 s of repetitive pulsing with high (1 Hz) and low (0.1 Hz) frequency under drug-free control conditions (squares) and in the presence of 3 μM ibogaine (circles). The pulse protocol was started 2 min after beginning of superfusion with bath solution or bath solution containing ibogaine. hERG tail current amplitudes were normalised to the drug-free control condition (data point at 60 s).

The concentration-dependence of hERG channel inhibition by ibogaine and 18-MC was determined as in our previous study (Koenig et al., 2012) by the pulse protocol shown in Fig. 2a, applied at a frequency of 1 Hz. An activating voltage step was followed by a 300 ms pulse to − 120 mV to elicit a large tail current. In untransfected cells no tail currents were observed at this potential. The peaks of the hERG tail currents were measured and plotted over time in Fig. 2b, which shows part of a typical experiment of drug application and wash out. We found that 1 and 10 μM ibogaine (filled bars) reversibly inhibit hERG currents in a concentration-dependent manner. Similarly, 10 μM of the ibogaine congener 18-MC (empty bar), reversibly inhibited hERG currents but to a somewhat lesser extent when compared to the same concentration of ibogaine (Fig. 2b). A summary of the concentration-dependencies of current inhibition by ibogaine and 18-MC from a series of such experiments (n = 4–17 and 4–13 for ibogaine and 18-MC, respectively) is shown in Fig. 2c. Normalised hERG tail current peaks, each time measured after a new steady-state level was reached during an external solution change, were plotted against drug concentration. Note that the ibogaine data were taken from Fig. 1b of our earlier study (Koenig et al., 2012; licence number, 2995391353547). Fits of the data points with a Hill equation revealed IC_50_ values of 3.9 ± 0.3 μM (Koenig et al., 2012) and 14.9 ± 0.8 μM (present study) for ibogaine and 18-MC, respectively. The corresponding Hill slopes were 0.8 and 0.9, and thus close to one, consistent with the presence of a single drug binding site.

Numerous drugs are known to increase their inhibition of hERG channels if the cell membrane is depolarised frequently, a phenomenon which is referred to as frequency-dependent block. To test if ibogaine inhibits hERG channels in a frequency-dependent manner, we first applied 3 μM ibogaine for 2 min to allow for a basal block without pulsing. This was followed by repetitive pulsing at low (0.1 Hz) and high (1 Hz) frequency for 60 s. The hERG tail current amplitudes normalised to the drug-free control condition (data point at 60 s) are shown in Fig. 2d. Upon repetitive pulsing a minor additional block developed at both frequencies. Thus, 60 s of 0.1 Hz and 1 Hz pulsing decreased the currents by an additional 15 ± 1% (n = 6) and 10 ± 7% (n = 7) compared to basal block levels, respectively. Consequently, 3 μM ibogaine does not generate considerable frequency-dependent hERG channel block.

### Drug effects on human Na_v_1.5 sodium channels heterologously expressed in TSA-201 cells

Besides hERG potassium channels, hNa_v_1.5 sodium channels are also of major importance in the human heart (Terrenoire et al., 2007). Since brain sodium channels were previously identified as one possible target site for ibogaine's actions (Glick et al., 2000), here we tested if ibogaine and its congener 18-MC also affect the currents through cardiac hNa_v_1.5 channels.

**Fig. 3:**
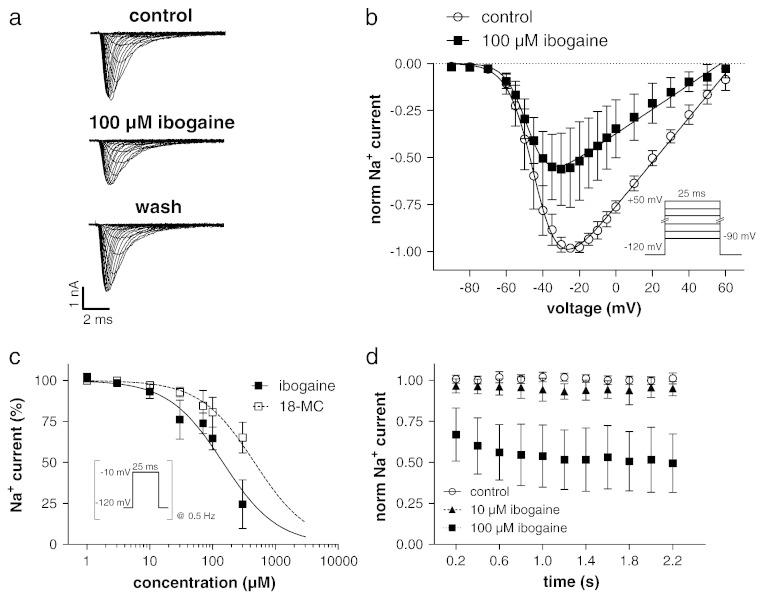
hNa_v_1.5 sodium channel blockade by ibogaine. (a) Typical currents through hNa_v_1.5 channels expressed in a TSA-201 cell under control conditions, after steady-state level was reached during superfusion with high sodium bath solution containing 100 μM ibogaine, and after wash out. The currents were elicited by the pulse protocol given in the inset of b. (b) Summary of the current–voltage relationships under control conditions and under inhibition with 100 μM ibogaine. The current peaks were plotted against the voltage. The solid lines represent best non-linear fits to the data points (function described in Materials and methods). (c) hNa_v_1.5 current inhibition by various concentrations of ibogaine and 18-MC. The pulse protocol to elicit the currents (inset) during the sequence of drug application and wash out was applied at a frequency of 0.5 Hz. The lines represent sigmoidal fits to the data points with a Hill equation; for 18-MC the slope was constrained to 1. (d) hNa_v_1.5 current reduction during repetitive high frequency (5 Hz) pulsing. The pulse protocol under experimental conditions (10 μM and 100 μM ibogaine) was always started 2 min after beginning of superfusion with bath solution containing the drug.

In Fig. 3a, typical whole cell currents through human Na_v_1.5 sodium channels heterologously expressed in a TSA-201 cell are shown under control conditions, after steady-state block with 100 μM externally applied ibogaine, and after wash out. The currents were elicited by the pulse protocol given in the inset of Fig. 3b. From a series of such experiments the inward current peaks were plotted against the applied voltage to obtain the current–voltage relationships (Fig. 3b). As can be observed in this figure and in the original traces of Fig. 3a, 100 μM ibogaine considerably reduced the sodium currents through hNa_v_1.5 channels. Moreover, in the presence of the drug, the current–voltage relation was slightly shifted to more hyperpolarized potentials. Thus, the voltage of half maximal activation (V_0.5_) was − 42 ± 5 mV in the absence and − 46 ± 5 mV in the presence of 100 μM ibogaine (p < 0.01, n = 7). The corresponding slope factors (K values) were 6.2 ± 0.8 and 5.5 ± 0.5 mV (p < 0.05), respectively. V_0.5_ after two minutes drug wash out was − 44 ± 4 mV, and K after wash out amounted to 6.7 ± 0.7 mV (n = 7).

The concentration-dependence of hNa_v_1.5 channel inhibition by ibogaine and 18-MC was determined by repetitive (0.5 Hz) 25 ms voltage steps to − 10 mV from a holding potential of − 120 mV (Fig. 3c). The elicited peak inward sodium current amplitudes were allowed to equilibrate until a stable condition had developed. Thereafter, different drug concentrations were washed in, and steady-state block level was determined. A complete wash out of the drug after each application served to confirm the control level before drug application. A summary of the concentration-dependent current inhibitions is shown in Fig. 3c. Fits of the data points with a Hill equation revealed IC_50_ values of 142 ± 11 μM (n = 5–10) and 464 ± 34 μM (n = 6–12) for ibogaine and 18-MC, respectively. The corresponding Hill slope was 1.1 for ibogaine, and it was constrained to 1 in case of 18-MC to give a reasonable estimation of the IC_50_ value, as 300 μM was the highest 18-MC concentration that could be applied (see Materials and methods).

Next we tested if hNa_v_1.5 channels are inhibited by ibogaine in a frequency-dependent manner. Therefore, either under control conditions or 2 min after start of superfusion with 10 or 100 μM ibogaine, repetitive high frequency pulsing (pulse protocol from Fig. 3c applied at 5 Hz) was started. The current peaks normalised to the respective control (drug-free condition) were plotted against time in Fig. 3d. As expected from the drug's concentration-dependence (Fig. 3c), 10 μM ibogaine generated only a minor basal block (see first data point). A considerable frequency-dependent decline in the current amplitude during repetitive 5 Hz pulsing could not be observed. Thus, the steady-state current level reached after 2 s of pulsing amounted to 96 ± 6% (n = 7) of the control (Fig. 3d). 100 μM ibogaine, on the other hand, inhibited the basal current to about 65% of the control, and generated a little further current reduction during repetitive pulsing (steady-state current level after 2 s, 52 ± 20% (n = 8); Fig. 3d). The corresponding steady-state current levels reached during repetitive 0.5 Hz pulsing were 93 ± 4% (n = 10) under 10 μM, and 65 ± 7% (n = 9) under 100 μM ibogaine.

Taken together, compared to hERG channel inhibition, significantly higher concentrations of ibogaine and 18-MC are needed to block hNa_v_1.5 sodium channels. This finding is in line with previous observations suggesting that many drugs have a higher affinity to hERG than to other cardiac ion channels (Perry et al., 2010).

### Ibogaine's effects on the action potential in adult ventricular guinea pig cardiomyocytes

After having characterised ibogaine's hERG channel inhibition and the drug's lower affinity inhibitory action on cardiac voltage-gated sodium channels, we next studied the effects of ibogaine on the cardiac action potential (AP). For this purpose we used cardiomyocytes isolated from adult guinea pig ventricles, which are considered an appropriate model system to study drug effects on the human cardiac AP (e.g. (Davie et al., 2004; Yao et al., 2008)).

**Fig. 4:**
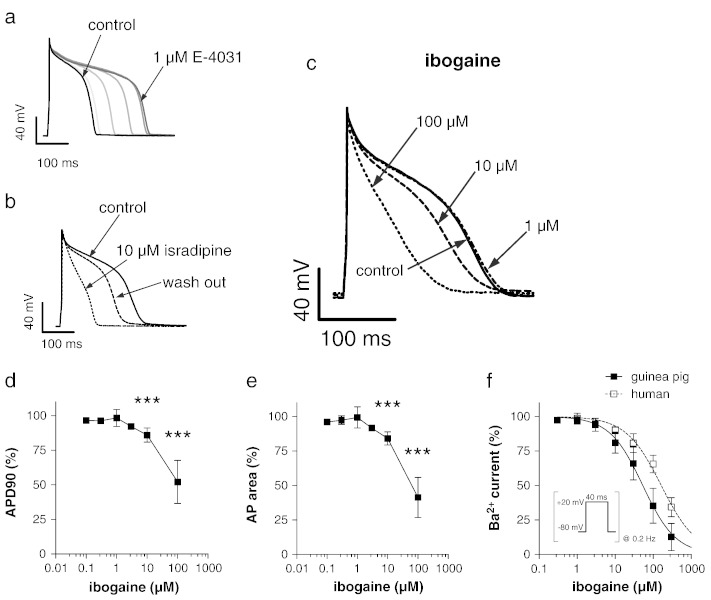
Effects of ibogaine on the action potential (AP) in guinea pig ventricular cardiomyocytes. During drug application and wash out experiments, APs were elicited at a frequency of 1 Hz. Ten consecutive traces were averaged for each depicted AP. (a) AP under control conditions, and at various time points after beginning of superfusion with bath solution containing 1 μM E-4031. The control is presented in black on the extreme left, whereas increasing grey scales represent the time points 100, 300, 500, 700 and 900 s after drug application. (b) AP under control conditions, 1 min after beginning of superfusion with bath solution containing 10 μM isradipine, and 3 min after wash out. (c) AP under control conditions, and each time after steady-state was reached during superfusion with bath solution containing 1, 10 and 100 μM ibogaine. The ibogaine effect on the AP was completely reversible after wash out (data not shown). (d) Concentration-dependence of the ibogaine effect on AP durations at 90% repolarisation (APD_90_). (e) Concentration-dependence of the ibogaine effect on the area under the AP. A significant difference existed among the different drug concentrations (p < 0.0001, ANOVA). *** indicates a significant difference to the drug-free control (p < 0.001, Tukey's post hoc test). The lines connect the data points. (f) Barium current inhibition by various concentrations of ibogaine in guinea pig cardiomyocytes (filled squares) and TSA-201 cells expressing hCa_v_1.2 channels (empty squares). The pulse protocol to elicit the currents during the sequence of drug application and wash out is shown in the inset. The lines represent sigmoidal fits to the data points with a Hill equation.

APs were recorded from single ventricular cardiomyocytes in the current-clamp mode. To validate our system, we first used the hERG channel blocker E-4031. As shown in Fig. 4a, externally applied E-4031 considerably prolonged the AP, as expected from a hERG channel blocker. Secondly, the l-type calcium channel blocker isradipine, which should shorten the AP, indeed reduced AP duration (Fig. 4b). After having performed these control experiments we tested the effects of ibogaine. Typical original traces under drug-free control conditions, and after steady-state level was reached following external application of ibogaine in different concentrations, are shown in Fig. 4c. It can be observed that 1 μM ibogaine did not considerably affect AP duration. This result was unexpected considering that this ibogaine concentration considerably blocks hERG channels (see Fig. 2). Furthermore, under 10 μM ibogaine AP duration was even significantly reduced, an effect that was dramatic under 100 μM drug concentration. Analyses of a series of such experiments (n = 5–21) on guinea pig cardiomyocytes are depicted in Figs. 4d and e. Here AP durations at 90% repolarisation (APD_90_, d), and the area under the AP (e) were plotted against ibogaine concentration. Both graphs suggest that low ibogaine concentrations (0.1–3 μM) do not alter AP duration. Interestingly, however, at a concentration of 1 μM, the drug occasionally generated recognisable AP prolongation. This effect was observed only at this particular ibogaine concentration, and only in two out of six tested cardiomyocytes, where it was reversible and appeared repeatedly after washout. Note that due to these two “outliers” the standard deviation for this data point (at 1 μM drug concentration) is abnormally large. Finally, higher ibogaine concentrations (≥ 10 μM) caused a significant shortening of the AP, as reflected by a decrease in the APD90 and AP area values (Figs. 4d and e). In an effort to better understand the lack of effects of ibogaine on the guinea pig AP despite showing potency for hERG, we studied the effects of the drug on the l-type calcium channel in more details. Thus, it seemed possible that a prominent inhibition of l-type calcium channels in guinea pig cardiomyocytes could counteract ibogaine's AP-prolonging effect generated by hERG channel blockade. Investigation of this issue in guinea pig cardiomyocytes (Fig. 4f) indeed revealed that ibogaine has a comparatively high affinity for calcium channels in this particular cell system (IC_50_, 53 ± 4 μM (n = 5–14); Hill slope, 1). Consequently, in guinea pig cardiomyocytes, 10 μM ibogaine inhibits the currents through calcium channels by approximately 20% (Fig. 4f), which may constitute a compensatory mechanism to counteract and even exceed the AP-prolonging effect of ibogaine's hERG channel blockade.

### Computer simulation to estimate the net effect of ibogaine's multiple ion channel inhibitory actions on the human AP

Several reports of QT interval prolongation after ibogaine intake in humans (Hoelen et al., 2009; Paling et al., 2012; Pleskovic et al., 2012) suggest that the drug, unlike in guinea pig ventricular cardiomyocytes, prolongs the AP in human cardiomyocytes. To test this hypothesis, we used the data already obtained with ibogaine on hERG and hNa_v_1.5, generated the data for hCa_v_1.2, and used these results to define the effects of ibogaine on a computer model of a human ventricular AP. hCa_v_1.2 expressed in TSA-201 cells was blocked by ibogaine with an IC_50_ value of 163 μM (Hill slope, 0.9; Fig. 4f), a value approximately 3-fold larger than that obtained in guinea pig cardiomyocytes.

**Fig. 5:**
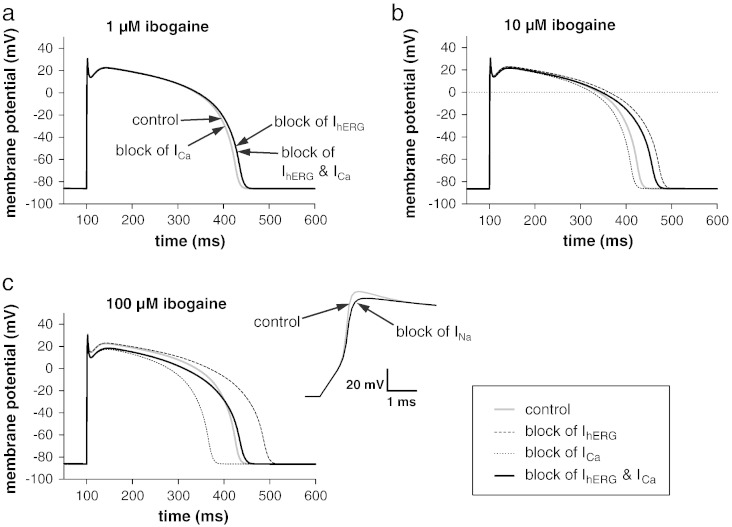
Computer simulation of the effects of ibogaine on the human cardiac AP at three different drug concentrations. In a and b, the effects of hERG and hCa_v_1.2 channel inhibition by the drug are considered both separately and together; the effect of hNa_v_1.5 channel inhibition was omitted, because at these ibogaine concentrations (1 and 10 μM) no effect on the AP was evident. In c, besides the effects of hERG and hCa_v_1.2 channel inhibition by the drug, the effect of hNa_v_1.5 channel inhibition on the upstroke phase of the AP is shown on an expanded time scale in the inset.

Fig. 5 shows the effects of hERG, hNa_v_1.5, and hCa_v_1.2 current inhibition on the AP at three different ibogaine concentrations. At 1 μM ibogaine, the simulations revealed that hERG current inhibition slightly prolongs the duration of the AP, when compared to the drug-free control condition (Fig. 5a). Calcium channel inhibition, on the other hand, did not generate any effect on the AP at this low drug concentration. At 10 μM ibogaine, hERG blockade considerably prolonged, and calcium channel blockade slightly shortened the AP, resulting in a net AP prolongation when both inhibitions were considered together (Fig. 5b). At both 1 and 10 μM ibogaine, hNa_v_1.5 current inhibition by the drug did not exert any noticeable effect on the AP. Therefore the respective traces were omitted. At the highest ibogaine concentration (100 μM) all ion channel inhibitions generated significant effects on the AP (Fig. 5c). First hERG blockade prolonged the AP. Secondly calcium channel blockade shortened the AP duration, and diminished the positive membrane potential reached during the plateau phase of the AP. Thirdly hNa_v_1.5 blockade slowed the upstroke and reduced the amplitude of the AP (inset of Fig. 5c). Finally, when hERG and hCa_v_1.2 channel inhibition at 100 μM ibogaine were considered together, hardly any effect on AP duration remained. Thus, at this high drug concentration, calcium channel inhibition obviously neutralised the effect produced by hERG channel blockade.

Taken together, the computer simulations revealed that low micromolar concentrations of ibogaine prolong the human cardiac AP due to hERG channel inhibition. The higher the ibogaine concentration becomes (already evident at 10 μM), the more calcium channel inhibition by the drug counteracts the AP-prolonging effect generated by hERG channel blockade.

### Human plasma protein binding of ibogaine

In an effort to define whether the concentrations used in this study could translate to reported clinical exposure, we determined the human plasma protein binding fraction of ibogaine using an equilibrium dialysis method (Cyprotex Discovery, Macclesfield, UK) and found it to be approximately 65%.

## Discussion

Here we report that low micromolar concentrations of ibogaine, a drug with promising anti-addictive properties, inhibit cardiac voltage-gated ion channels. The strongest potency of ibogaine we found here relates to hERG channels, and the drug concentration required to inhibit hERG currents by 50% (IC_50_) was 4 μM (Koenig et al., 2012; present study). Ibogaine concentrations have been measured in whole blood samples of humans after single oral doses of 500–1000 mg (Mash et al., 1998, 2000), doses that are typically employed to treat drug addicts (10–25 mg/kg of body weight) (Alper, 2001; Mash et al., 1998, 2000), and in a case of ibogaine poisoning (Kontrimaviciute et al., 2006). The values obtained were 1–10 μg/ml (3–30 μM) and represent total drug concentrations. With the extent of ibogaine's human plasma protein binding of 65% taken into account, the free plasma concentrations reached after drug intake in these studies amounted to 1–11 μM. Together this suggests that therapeutic concentrations of ibogaine directly inhibit hERG channels in the human heart.

### Ibogaine's multiple ion channel inhibitory actions: prolongation or shortening of the cardiac AP?

hERG channel blockade by drugs is known to delay the repolarisation phase of the cardiac AP, which usually goes along with a prolongation in AP duration. Here, although confirming ibogaine's inhibitory effect on hERG channels (Koenig et al., 2012), surprisingly, we did not observe the expected AP prolongation in guinea pig cardiomyocytes by ibogaine in any of the concentrations (range, 0.1–100 μM) used. On the contrary, higher ibogaine concentrations (≥ 10 μM) even shortened AP duration significantly. To reconcile this discrepancy, here we propose that ibogaine's effects on l-type calcium channels, which normally depolarise the membrane potential when activated, counteract AP prolongation due to ERG channel inhibition by the drug. This is supported by the fact that low micromolar ibogaine concentrations inhibit the currents through calcium channels in guinea pig cardiomyocytes. Thus at 10 μM ibogaine (minimum drug concentration required to induce AP shortening), the currents through calcium channels are already reduced by approximately 20% (Fig. 4f; IC_50_, 53 μM). In guinea pig cardiomyocytes ibogaine seems to display a profile similar to the calcium channel blocker verapamil. Verapamil inhibits both hERG and calcium channels at therapeutic concentrations and does not induce a prolongation of the cardiac AP (e.g. (Redfern et al., 2003)). On the other hand, when implementing both ibogaine's human ERG and Ca_v_1.2 channel inhibitions in a computer model of a human cardiac AP, the drug produced considerable AP prolongation at certain concentrations (Fig. 5). Here, e.g. at 10 μM, ibogaine's inhibitory effect on hCa_v_1.2 channels (Fig. 4f; IC_50_, 163 μM) only partly counteracts its hERG channel blocking effects.

Together these findings strongly suggest that calcium channel blockade by ibogaine counteracts the AP-prolonging effect of ERG channel inhibition by the drug. This effect occurs more prominently in guinea pig (experimental data) compared with human (computer simulation) cardiomyocytes, probably due to the 3-fold higher affinity of ibogaine for guinea pig versus human calcium channels. As a consequence, only the human, but not the guinea pig cardiac AP is prolonged by ibogaine in low micromolar concentrations. Species differences in drug affinities for ion channels, as observed for the effects of ibogaine on l-type calcium channels in the present study, have previously been reported (e.g. (Grace and Camm, 2000)). Because in the present study we did not have the possibility to measure APs in human ventricular cardiomyocytes but performed computer simulations instead, however, these interpretations remain speculative.

It should be noted here that also other factors may have contributed to the different actions of ibogaine on the AP in guinea pig and human. First, the drug may have a lower affinity for the guinea pig compared with the human ERG channel. Secondly, the contribution of ERG currents to the repolarisation phase of the cardiac AP may be larger in human compared to guinea pig hearts (O'Hara and Rudy, 2012; Zicha et al., 2003). Thus, in the latter, a more significant part of the repolarisation may be carried by the slow delayed rectifier potassium current IK_s_, which is strongly expressed in guinea pig ventricular cardiomyocytes (Sanguinetti and Jurkiewicz, 1990). Consequently, ERG channel blockade by ibogaine would have a stronger AP-prolonging effect in human than in guinea pig cardiomyocytes. Finally, we can also not exclude the possibility that ibogaine additionally affects other cardiac ion channels than ERG and calcium channels, which are expressed in guinea pig cardiomyocytes, but were not considered in our human AP simulations.

### Does ibogaine contribute to QT interval prolongation in humans?

Ibogaine is known to affect the cardiovascular system. The drug slows the heart rate in animals (Binienda et al., 1998; Glick et al., 2000) and humans (Alper, 2001; Glick et al., 2000; Maciulaitis et al., 2008). This effect is probably mediated via interaction of ibogaine with muscarinic acetylcholine receptors (Alper, 2001; Glick and Maisonneuve, 2000; Glick et al., 2000). Moreover, cardiac arrhythmias have been related to several cases of sudden death after ibogaine intake (Alper et al., 2012; Hoelen et al., 2009; Maas and Strubelt, 2006). However, due to concomitant medications used and comorbidities (e.g. cardiovascular) present in the patients described in these cases (Alper et al., 2012; Donnelly, 2011), it is unclear whether indeed ibogaine has caused these deaths. First direct evidence for ibogaine-induced arrhythmias was provided by Hoelen et al. (2009). These authors reported a severe prolonged QT interval of the electrocardiogram, associated with ventricular tachyarrhythmias, in a 31-year-old woman after she had taken a single dose of ibogaine. Other cases of ibogaine-associated QT interval prolongation and ventricular arrhythmias were recently published (Paling et al., 2012; Pleskovic et al., 2012).

In the present study, we describe both ibogaine effects on cardiac ion channels that favour (hERG channel inhibition) and counteract (calcium channel inhibition) AP prolongation. Because ibogaine's affinity for human ERG channels is considerably higher (40-fold) than that for human Ca_v_1.2 channels, we propose that the drug can prolong AP duration in human ventricular cardiomyocytes, and thereby has the propensity to prolong the QT interval. It should be noted here, however, that although most probably insufficient to fully counteract AP prolongation due to hERG channel inhibition, calcium channel blockade will in any case diminish the amount of QT interval prolongation generated by the drug (e.g. at 10 μM ibogaine, see Fig. 5b). Here, another consideration is noteworthy: with the exception of the patient recently described in Pleskovic et al. (2012), the other reported cases of QT interval prolongation after ibogaine intake in humans (Hoelen et al., 2009; Paling et al., 2012) were all accompanied by hypokalaemia. Hypokalaemia has been shown to reduce the cell surface density of hERG channels, and exacerbates long QT syndrome (Guo et al., 2009). Thus, it cannot unequivocally be judged, whether the cases of QT prolongation reported (Hoelen et al., 2009; Paling et al., 2012) can solely be attributed to the direct inhibitory effect of ibogaine on hERG channels in these patients. Moreover, low extracellular potassium may also increase drug blockade of IK_r_ (Yang and Roden, 1996). If this is also true for ibogaine, hypokalaemia may enhance the direct QT prolonging effect of the drug via hERG channel inhibition. Finally, besides QT prolongation as a possible consequence of hERG inhibition, it should be noted that the application of a powerful psychoactive drug such as ibogaine may also lead to cardiac adverse effects related to its central nervous activity without the need to imply hERG inhibition. Regardless of the actual mechanism underlying the potential ibogaine-induced QT prolongation, this condition may likely be detrimental for humans at high risk such as in the case of drug addicts.

### Is the ibogaine congener 18-Methoxycoronaridine safer than ibogaine?

In this study, besides ibogaine, we also tested its synthetic congener 18-MC, which is believed to be less toxic (Glick et al., 1996, 2000, 2001, 2011). We found that, similar to ibogaine, 18-MC inhibits hERG and hNa_v_1.5 channels heterologously expressed in TSA-201 cells. For the inhibition of both these currents, however, higher concentrations of 18-MC were needed when compared to ibogaine. This suggests that the affinity of 18-MC to cardiac voltage-gated ion channels is lower than that of ibogaine. In accord, Glick et al. (2000) reported that 18-MC has a lower affinity for rat brain sodium channels than ibogaine. In principle, the lower affinities of 18-MC for cardiac ion channels suggest a reduced risk for cardiac adverse effects in comparison with ibogaine. However, 18-MC's affinity for hERG channels (IC_50_, 15 μM) is still close to the therapeutic concentration range (see above), if similar plasma protein binding as for ibogaine is assumed. Thus, like ibogaine, 18-MC may have the propensity to induce QT interval prolongation by hERG channel inhibition. 3- to 4-fold higher concentrations of 18-MC, however, may be needed to trigger this effect. Finally, it should be mentioned that a racemate of 18-MC was used here for testing. This may restrict the conclusions drawn, because single enantiomers could possess different ion channel activities.

### Limitations of the study

The current inhibition data used for the human AP computer simulations originated from human ion channels (hERG, hNa_v_1.5, and hCa_v_1.2) heterologously expressed in TSA-201 cells. Drug affinities for ion channels in native cells can differ from those detected in heterologous expression systems. Thus, our simulations can only provide an estimate of the real situation in native human cardiomyocytes. This certainly limits our predictions about ibogaine's effects on the heart's electrophysiology in humans. We are also aware that, by assessing the cardiac ion channel profile of ibogaine (and 18-MC), we did not directly test proarrhythmia liability or vulnerability.

In conclusion, we found that ibogaine inhibits cardiac voltage-gated ion channels. At therapeutic concentrations, the drug's inhibitory effect on hERG channels provides a potential explanation for the reported QT interval prolongation in humans that may lead in some cases to tachyarrhythmias. Ibogaine derivatives with reduced propensity to block cardiac ion channels but preserved anti-addictive properties need to be developed. 18-MC may be regarded as a first candidate.

## Conflict of interest

The authors declare that there are no conflicts of interest.
